# Explanation Wanted

**Published:** 1889-02

**Authors:** P. P. Wells

**Affiliations:** Brooklyn


### EXPLANATION WANTED.

DEAR HOMŒOPATHIC PHYSICIAN:—In your issue for January, 1889, p. 25, I find my friend Dr. Julius Schmitt saying:

"I do not agree with Dr. P. P. Wells, that a high potency will act if a low will."

As I do not remember having said anything like this, and am certain I never thought it, will Dr. S. kindly give the time and place where he understood me to have said this?

I have said several times, and the last time, I believe, was in June last, and the place Niagara Falls, that I had in my own homœopathic practice of more than forty-five years, never gained for my patient, by going from a higher to a lower potency. At the same time I believe I added, as I have been accustomed to do, "I have neighbors of intelligence and veracity who have had a different experience, and I accept their testimony as to their gain from low numbers after the high. This does not sound like the utterance attributed to me by Dr. S. Will he explain?

P. P. WELLS.

BROOKLYN, January 9th, 1889.

